# Draft genomes of three *Enterococcus* species recovered from the gastrointestinal microbiota of healthy domestic rabbits (*Oryctolagus cuniculus*)

**DOI:** 10.1128/mra.01162-25

**Published:** 2026-03-04

**Authors:** Abimbola Olumide Adekanmbi, Oluwatosin Olubunmi Oladipo, Opeyemi U. Lawal, Valeria R. Parreira, Bolaji Fatai Oyeyemi, Olatunji Abubakar Jimoh, Aderemi Akinyemi, Charles Ayorinde Ologunde, Ayoponsi Bukola Olaoye, Olugbenga David Oloruntola, Mitra Soni, Harmanpreet Kaur, Lawrence Goodridge

**Affiliations:** 1 Environmental Microbiology and Biotechnology Laboratory, Department of Microbiology, University of Ibadan 58987, Ibadan, Nigeria; 2 Molecular Biology and Biotechnology Laboratory, Department of Microbiology, University of Ibadan 58987, Ibadan, Nigeria; 3 Science Technology Department, The Federal Polytechnic Ado-Ekiti, Ado-Ekiti, Ekiti State, Nigeria; 4 School of the Environment, University of Windsor 8637, Windsor, Canada; 5 Great Lakes Institute for Environmental Research, University of Windsor 8637, Windsor, Canada; 6 Canadian Research Institute for Food Safety, University of Guelph 3653, Guelph, Ontario, Canada; 7 Cell Biology and Genetics Unit, Department of Zoology, University of Ilorin 108285, Ilorin, Nigeria; 8 Department of Agricultural Technology, The Federal Polytechnic Ado-Ekiti, Ado-Ekiti, Ekiti State, Nigeria; 9 Department of Animal Science, Adekunle Ajasin University 107994, Akungba Akoko, Nigeria; Nanchang University, Nanchang, Jiangxi, China

**Keywords:** genome sequence, *Enterococcus*, domestic rabbit

## Abstract

We report the draft genome sequences of three *Enterococcus* strains isolated from the gastrointestinal tract of domestic rabbits. These genomes contribute to expanding the representation of *Enterococcus* diversity in non-conventional hosts and provide resources for future comparative and functional genomics of gut-adapted Enterococci.

## ANNOUNCEMENT

The genus *Enterococcus* comprises gram-positive, facultatively anaerobic bacteria commonly found in the gastrointestinal tracts of humans and animals (1, 2). While *Enterococcus faecalis* (*E. faecalis*) and *Enterococcus faecium* (*E. faecium*) are well-characterized due to their clinical relevance and prevalence in human microbiota, enterococci from non-human hosts, particularly lagomorphs, remain understudied (3). Rabbits possess a distinct hindgut-fermenting digestive system, and their gut microbiota harbors unique microbial lineages shaped by herbivorous diet and cecotrophy (4, 5). Here we present the draft genomes of three *Enterococcus* isolates recovered from healthy Hyla rabbits (crossbreed of California, New Zealand, and Chinchilla rabbits), predominant in Nigeria and intended for meat production. These isolates were sequenced as part of the Global Bacterial Antibiotic Resistance Sequencing (GLOBAR-SEQ) program.

One gram of the gastrointestinal content of an individual rabbit was serially diluted in 9 mL of sterile distilled water, plated on de Man, Rogosa, and Sharpe (MRS) medium, and incubated anaerobically at 35 ± 2°C for 48 h. Distinct colonies presumptive of lactic acid bacteria (LAB) were subcultured on MRS plates to obtain pure cultures. Preliminary identification was performed using the VITEK 2 Compact System (bioMérieux, Inc., Canada). Genomic DNA was extracted from three selected colonies using the DNeasy Blood and Tissue Kit (Qiagen) according to the manufacturer’s and as previously described (6). DNA libraries were prepared using the Illumina DNA prep tagmentation kit (#20018704) and IDT for Illumina DNA/RNA UD indexes (#20027213) following the manufacturer’s instructions. Paired-end (2 × 150 bp) sequencing was performed using the high-output flow cell on the Illumina NextSeq 1000 instrument.

Overall, 3,405,927 sequencing reads were obtained on average. Raw reads were quality filtered using FastQC v0.11.9 (https://github.com/s-andrews/FastQC), and trimmed using Trimmomatic v0.39 (7). Reads with Phred scores of >20 were assembled *de novo* using Skesa v2.4.0 (8). Assembly quality was assessed using QUAST v5.2 (9). Genome completeness and contamination were evaluated with CheckM v1.2.2. (10). Taxonomic assignment was refined using GTDB-Tk v2.3.0 with GTDB release 214 (11). Genome annotation was performed using the NCBI Prokaryotic Genome Annotation Pipeline v6.3 (12), and sequence type was determined using PubMLST (13). Default parameters were used for all bioinformatics tools.

The Whole Genome Sequence-based approach identified the three LABs as *Enterococcus lactis* (*E. lactis*) UIADO22, *Enterococcus hirae* (*E. hirae*) UIADO30, and *E. lactis* UIADO37. The draft genome sizes of the isolates ranged from 2,942,513 to 2,990,747 bp, with an average of 33 contigs. GC contents were comparable (38.05%) in both *E. lactis* isolates and slightly lower in *E. hirae* (36.69%). The predicted protein coding sequences ranged from 2,683 in *E. hirae* to 2,757 in *E. lactis* UIADO22.

Other assembly statistics, including N50 values, tRNA counts, and genome completeness and contamination, are presented in Table 1. Taken together, these draft genomes provide foundational resources for comparative studies on host adaptation, commensalism, and opportunistic potential within the genus.

**TABLE 1:** Summary of sequence metrics of the genomes of the three *Enterococcus* spp.

Features/identity	*E. lactis* UIADO22	*E. hirae* UIADO30	*E. lactis* UIADO37
SRA accession No.	SRR34113111	SRR34113110	SRR34113109
Assembly accession No.	JBRNWW000000000	JBRNWV00000000	JBRNWU000000000
No. of Reads	3,441,322	3,360,262	3,416,198
Genome size (bp)	2,942,513	2,990,747	2,942,524
No. of contigs	37	29	34
Contig N50	313,623	202,048	439,901
Contig L50	3	5	3
GC content (%)	38.05	36.69	38.05
CDS (with protein)	2,757	2,683	2,752
Genome completeness (%)	100	100	100
CRISPR arrays	0	1	0
tRNA	57	57	57
Sequence type	329	Not assigned	329

## Data Availability

This Whole Genome Shotgun project has been deposited at DDBJ/ENA/GenBank under the accessions PRJNA1281047.

## AFTER EPUB

[This article was published on 4 March 2026 with an error in Table 1. The Table 1 was corrected in the current version, posted on 26 March 2026.]
